# The Independence of Signaling Pathways Mediating Increased Expression of Plasminogen Activator Inhibitor Type 1 in HepG2 Cells Exposed to Free Fatty Acids or Triglycerides

**DOI:** 10.1080/15604280214488

**Published:** 2002

**Authors:** Yabing Chen, David J. Schneider

**Affiliations:** 1 Department of Medicine The University of Vermont Burlington VT 05405 USA; 2 College of Medicine University of Vermont 208 South Park Drive Suite 2 Colchester VT 05446 USA

## Abstract

We have shown that both free fatty acids (FFA) and triglycerides (TG) increase expression of plasminogen activator inhibitor type 1 (PAI-1) in vivo and in vitro. To determine signaling mechanisms responsible, HepG2 cells were exposed to FFA, emulsified TG, or the combination. The combination of FFA and TG increased PAI-1 to a greater extent than either agent alone (fold induction: 0.45mM FFA 1.7±0.2, 1000mg/dl TG 1.9±0.1, both 2.3±0.2, n=10, p<0.05 for comparison of combination with either alone). Cells transfected with PAI-1 5' flanking region containing the 4G or 5G polymorphism displayed similar activity in response to FFA, but modestly greater activity with the 4G polymorphism in response to TG (fold induction: 5G-1.28±0.14 and 4G- 1.46±0.13, n=6, p<0.05 for comparison). Deletion analyses demonstrated that FFA and TG induce PAI-1 expression through distinct regions of the promoter. Inhibition of protein kinase C inhibited the response to FFA but not TG. Accordingly, increased FFA and TG contribute to increased PAI- I through independent mechanisms.


*Int. Jnl. Experimental Diab. Res.*, 3:109-118, 2002
© 2002 Taylor and Francis
1560-4284/02 $12.00 + .00

### The Independence of Signaling Pathways Mediating Increased Expression of Plasminogen Activator Inhibitor Type 1 in HepG2 Cells Exposed to Free Fatty Acids or Triglycerides

YABING CHEN AND DAVID J. SCHNEIDER*

Department of Medicine, The University of Vermont, Burlington, VT 05405, USA

*Received: September 28, 2001; In final form: December 20, 2001*

We have shown that both free fatty acids (FFA) and triglycerides (TG) increase expression of plasminogen activator inhibitor type 1 (PAI-1) in vivo and in vitro. To determine signaling mechanisms responsible, HepG2 cells were exposed to FFA, emulsified TG, or the combination. The combination of FFA and TG increased PAI-1 to a greater extent than either agent alone (fold induction: 0.45mM FFA 1.7±0.2, 1000mg/dl TG 1.9±0.1, both 2.3±0.2, n=10, $p<0.05$ for comparison of combination with either alone). Cells transfected with PAI-1 5' flanking region containing the 4G or 5G polymorphism displayed similar activity in response to FFA, but modestly greater activity with the 4G polymorphism in response to TG (fold induction: 5G-1.28±0.14 and 4G-1.46±0.13, n=6, $p<0.05$ for comparison). Deletion analyses demonstrated that FFA and TG induce PAI-1 expression through distinct regions of the promoter. Inhibition of protein kinase C inhibited the response to FFA but not TG. Accordingly, increased FFA and TG contribute to increased PAI- I through independent mechanisms.

*Key words:* diabetes, free fatty acids, triglycerides, plasminogen activator inhibitor type 1, protein kinase C

*Corresponding author: College of Medicine, University of Vermont, 208 South Park Drive, Suite 2, Colchester, VT 05446; e-mail: djschnei@zoo.uvm.edu; tel: (802) 847-3734; fax: (802) 656-8969

### INTRODUCTION

Cardiovascular disease is the leading cause of death in patients with diabetes, particularly type 2 diabetes. Patients with type 2 diabetes have increased concentrations of PAI-1 in blood and in vessel walls [1, 2, 3, 4]. PAI-1 is the primary physiologic inhibitor of plasminogen activators, and increased expression of PAI-1 is likely to accelerate atherogenesis and contribute to an increased incidence of acute myocardial infarction. Thus, identification of mechanisms responsible for increased expression of PAI-1 should facilitate development of approaches to treatment designed to decrease expression of PAI-1, retard progression of atherosclerosis, and prevent myocardial infarction.

Both hormonal and metabolic abnormalities contribute to increased expression of PAI-1 in diabetes. The concentrations in blood of triglycerides (TG) and free fatty acids (FFA) correlate positively with those of PAI-1 [1, 2, 5, 6, 7, 8, 9, 10]. We have reported direct effects of TG and FFA on expression of PAI-1 *in vivo* and *in vitro* [11, 12]. PAI-1 increases in blood from healthy human subject during infusion of glucose and emulsified TG (in combination with heparin to increase FFA concentrations). We and others have shown that both TG and FFA increase expression of PAI-1 in human hepatoma (HepG2) cells [5, 12, 13, 14, 15, 16, 17, 18, 19].

VLDL has been shown to augment expression of PAI-1 in endothelial cells by increasing the binding of a VLDL-inducible transcription factor to the PAI-1 promoter [13, 14]. We have

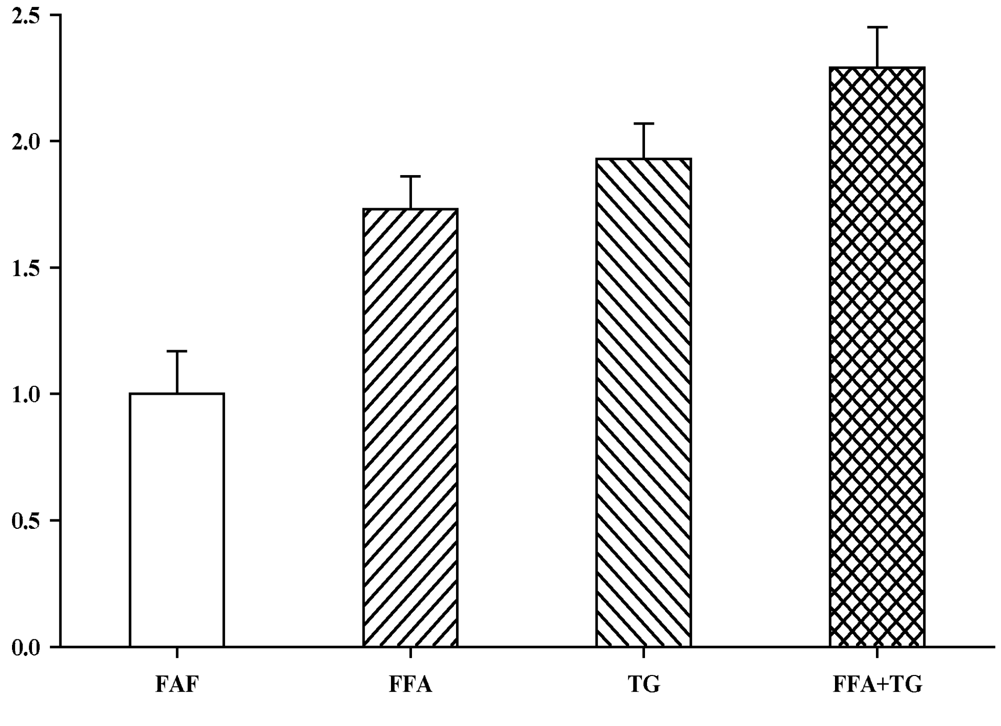

FIGURE 1
FFA and TG augmented accumulation of PAI-1 in media conditioned by HepG2 cells. HepG2 cells (80% confluent) were exposed to serum-free DME/F12 for 8 hours and subsequently to fatty acid free BSA, 3% BSA (0.45mM FFA), 1000mg/dl Liposyn® or the combination of FFA and TG for 24 hours. The concentrations of PAI-1 were determined by ELISA in conditioned media. Values are means ± SD and represent results in 10 determinations in each case ($p < 0.001$ compared with control, $p < 0.05$ for the combination compared with either treatment alone).

recently identified a FFA response region in human PAI-1 5' flanking DNA that is distinct from the VLDL response region [16]. Because VLDL contains both TG and FFA, the present studies were designed to determine whether FFA and TG augment expression of PAI-1 through independent signaling pathways.

### MATERIALS AND METHODS

### *Cell culture*

HepG2 cells were obtained from American Type Culture Collection and grown in minimal essential media (MEM, Gibco-BRL) with 10% Nuserum (Gibco-BRL). Experiments were performed in Dulbeccos' modified Eagles media with Hams' nutrient mixture F12 (DME-F12, Gibco-BRL).

### *FFA and TG preparation and treatment*

3% bovine serum albumin (BSA) and selected concentrations of TG (50mg/dl, 100mg/dl, 500mg/dl and 1000mg/dl Liposyn®) were prepared in DME/F12. 3% BSA containing media were adjusted to pH 7.4 and filtered through a 0.22 mm filter. We found that 3% BSA in DME/F12 contains 0.45 mM FFA (Wako NEFA C kits, Biochemical Diagnostics). 80% confluent HepG2 cells were pre-incubated in serum-free DME/F12 for 4 to 8 hours. Subsequently, experiments were performed in DME/F12 with FAF-BSA, DME/F12 with BSA, or DME/F12 with Liposyn®.

### *Protein Kinase C (PKC) inhibitor*

H-7 (Calbiochem), a broad based serine-threonine kinase inhibitor and Bisindolylmaleimide I (Calbiochem), a highly selective cell-permeable PKC inhibitor (Ki=10nM) were added to cells exposed also to FFA or TG. Both H-7 and Bisindolymaleimide I were dissolved in DME/F12.

### *Detection of PAI-1 antigen*

Conditioned media were collected 24 hours after treatment and assayed for PAI-1 by ELISA. (Tintelize, Biopool).

### *Construction of PAI-1 Promoter-luciferase Reporter Plasmids*

PAI-LUC 1313 and PAI-LUC 328, containing 1313 bp or 328 bp of 5'-flanking (-1313) and 74 bp of the untranslated first exon (+74) of human PAI-1 DNA upstream of the luciferase reporter in plasmid pGL3-Basic (Promega), were generated as described [16]. A 72 bp fatty acid response region (-599 to -528) was deleted from PAI-LUC 1313 to make PAI-LUC 1313-72. PAI-LUC 1313-120 was generated by deletion of a 120 bp Pml I fragment (-680 to -560) from PAI-LUC 1313.

PAI LUC 4G was generated by replacing the 5G region in PAI LUC 1313 with 4G containing fragment by PCR with primers synthesized by Sigma Genosys: 5'-tgg tca cgt ggg gag tca gcc gt-3' and 5'-tgg tgg agg tcc ttt ctc-3'. The sequence was confirmed by sequence analysis.

### *Transient transfection*

HepG2 cells were transfected with the use of the calcium-phosphate precipitation method [16]. pRL-CMV, a plasmid with Renilla luciferase gene downstream of the CMV promoter, was used to assess transfection efficiency. Luciferase activity was detected in cell extracts with the Passive Lysis Buffer in the Dual-Luciferase Reporter Assay System (Promega). Luciferase activities were determined by Dual-Luciferase Reporter Assays with a DLReady luminometer (Turner Designs Instrument).

### *Statistical Analysis*

Results are means ± SD. Differences between two groups were identified with Student's t tests. For multiple groups one-way analysis of variance and Student-Newman-Keuls Tests

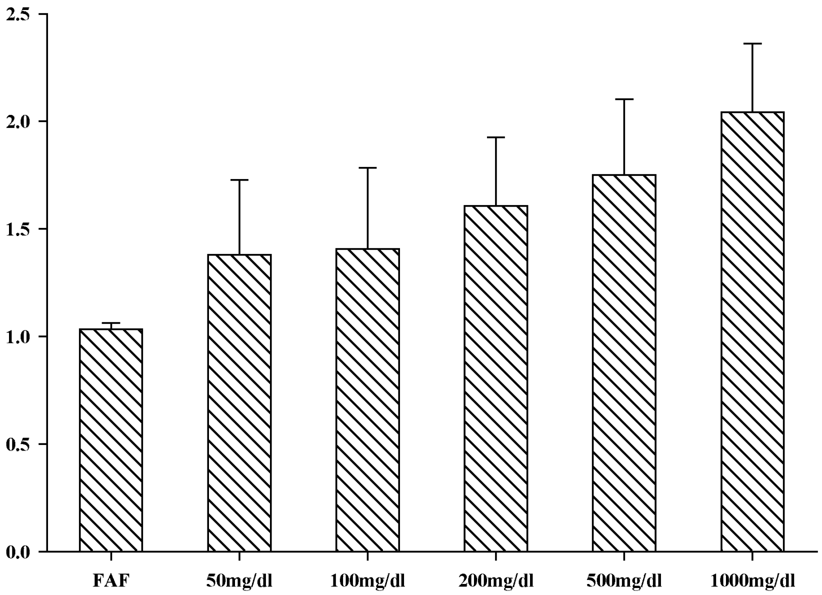

FIGURE 2
TG increased 24-hour accumulation of PAI-1 in a dose-dependent manner. HepG2 cells (80% confluent) were exposed to serum-free DME/F12 for 8 hours and subsequently to selected concentrations of Liposyn® for 24 hours. The concentration of PAI-1 was determined by ELISA in conditioned media. Values are means ± SD of 6 determinations in each case ($p < 0.05$ for 50mg/dl and 100mg/dl and $p<0.001$ for 200mg/dl and greater compared with control).

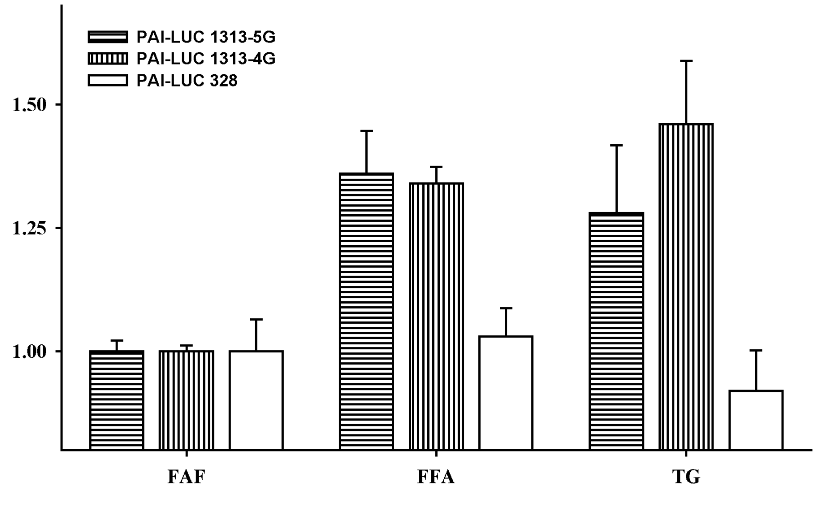

FIGURE 3
Luciferase activity in proteins extracted from HepG2 cells transfected with 1313 bp of the 5' flanking DNA containing the 4G or 5G polymorphism at -675. Transient transfection of HepG2 cells was performed with the use of calcium-phosphate precipitation. HepG2 cells were co-transfected with PAI-LUC constructs and pRL-CMV, a plasmid containing a Renilla luciferase gene downstream of the CMV promoter, to control for transfection efficiency. Cells were incubated in serum-free media for 8 hours and then exposed to fatty acid free (FAF) BSA, 3% BSA (0.45mM FFA), or 1000mg/dl Liposyn® in DMEM/F12 for 24 hours. HepG2 cell extracts were prepared with Passive Lysis Buffer in the Dual-Luciferase Reporter Assay System and luciferase activities were determined with the Dual-Luciferase Reporter Assay on a DLReady luminometer. Results are the means ± SD of 6 determinations ($p<0.001$ for the PAI-LUC 1313 constructs compared with control, $p<0.05$ for the PAI-LUC 1313-4G compared with PAI-LUC 1313-5G after exposed to TG).

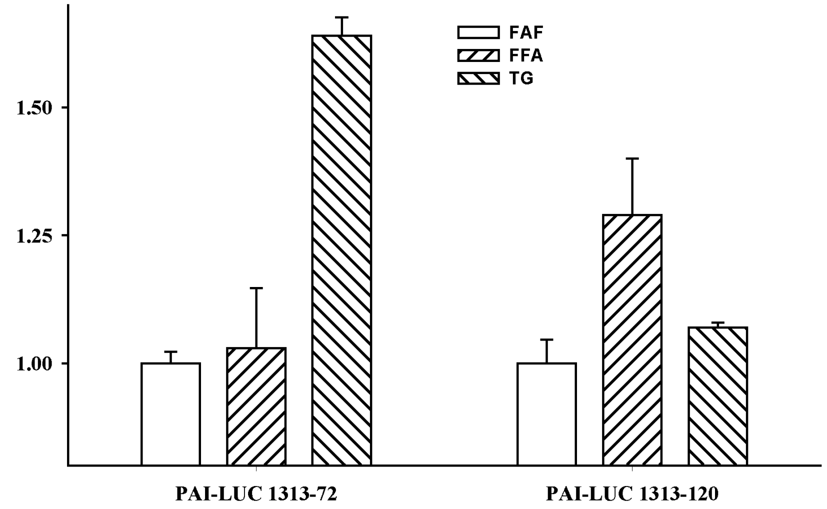

FIGURE 4
Deletion analysis of the PAI-1 promoter. HepG2 cells were transiently transfected with PAI-1 promoter constructs PAI LUC 1313-72 (deletion of -528 to –599 from PAI LUC 1313) or PAI LUC 1313-120 (deletion of -680 to –560 from PAI LUC 1313). The Renilla Luciferase reporter pRL-CMV was co-transfected to control for transfection efficiency. Cells were incubated in serum-free media for 8 hours and then exposed to fatty acid free BSA (FAF-BSA), 3% BSA (0.45mM FFA) or 1000 mg/dl Liposyn® for 24 hours. Luciferase activity was determined by Dual-Luciferase Reporter Assay. Results are mean ± SD of 6 determinations ($p<0.05$ for PAI-LUC 1313-72 when TG was FFA or control, and for PAI-LUC 1313-100 when FFA was compared with TG or control).

were used to identify differences. Significance was defined as $p < 0.05$.

### RESULTS

### *FFA and TG Increase Expression of PAI-1*

Both FFA (3% BSA) and TG (1000mg/dl Liposyn®) increased 24-hour accumulation of PAI-1 (Figure 1). Similar to previously reported results with FFA [16], a concentration dependent increase in 24-hour accumulation of PAI-1 was seen after exposure of cells to TG (Figure 2). The combination of FFA and TG yielded greater effects than either agent alone (Figure 1, n=10, $p<0.05$ for combination compared with each agent alone).

### *FFA and TG Increase Luciferase Activity*

Both FFA and TG increased luciferase activity (corrected for transfection efficiency) in extracts from HepG2 cells transfected with a chimeric gene with 1313 bp of human PAI-1 5' flanking DNA upstream of a luciferase reporter (PAI-LUC 1313) but not with PAI-LUC 328 (328 bp of human PAI-1 5' flanking DNA upstream of a luciferase reporter, Figure 3).

### *Differential Effects of the 4G and 5G polymorphism*

Luciferase activity was increased similarly when cells were transfected with PAI-LUC 1313 containing the 4G or 5G polymorphism (at -675 in the PAI-1 gene) and exposed subsequently to FFA (Figure 3). Cells transfected with PAI-LUC 1313 containing the 4G polymorphism exhibited modestly greater luciferase

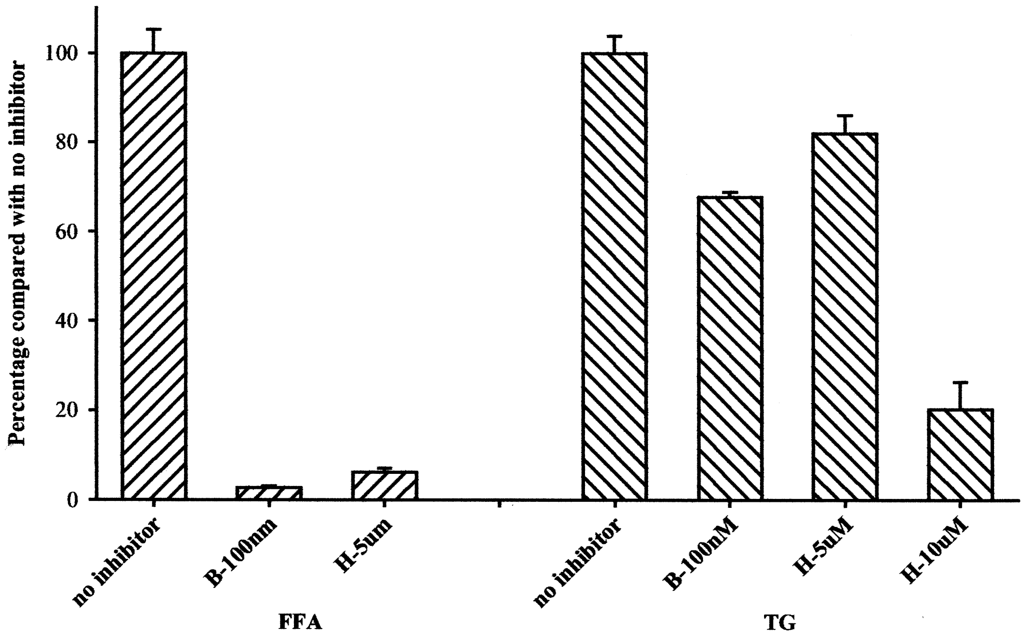

FIGURE 5
The Effect of an inhibitor of Protein Kinase C (Bisindolylmaleimide I, [B]) and that of a broad serine-threonine kinase inhibitor (H-7, [H]) on the increased expression of PAI-1 induced by TG and FFA. 80% confluent HepG2 cells were exposed to serum free DME/F12 for 8 hours and then treated simultaneously with indicated concentrations of the inhibitors plus either 3% BSA (0.45mM FFA), or 1000mg/dl Liposyn®. Conditioned media were collected 24 hours later, and the concentration of PAI-1 was determined by ELISA. Values are means ± SD of 8 determinations in each case ($p<0.001$ for no inhibitors compared with inhibitors in response to FFA and TG).

activity in response to TG (fold induction with 1000 mg/dl Liposyn® 1.28 ± 0.14 (with the 5G polymorphism) and 1.46 ± 0.13 (with the 4G polymorphism), figure 3, n=6, $p<0.05$ for the comparison of 5G to 4G).

### *PAI-1 Promoter Deletion Analysis*

Deletion of a 72 bp segment (-528 to -599) from PAI-LUC 1313 abolished the response to FFA but did not affect the response to TG (Figure 4). Deletion of a 120 bp segment (-680 to -560) from PAI-LUC 1313 abolished the response to TG but did not affect response to FFA (Figure 4).

### *FFA, TG and Protein Kinase C activity in HepG2 Cells*

Both H-7 (H), a broad based serine-threonine kinase inhibitor, and Bisindolylmaleimide I (B), a highly selective cell-permeable protein kinase C inhibitor (Ki=10 nM), inhibited the FFA-induced increased expression of PAI-1 in HepG2 cells by more than 90%. By contrast, these agents inhibited the increased expression of PAI-1 induced by TG by only 30% (Figure 5). Greater concentrations of H-7 attenuated the TG-induced increased expression of PAI-1 to a greater extent (Figure 5).

### DISCUSSION

Elevated concentrations of PAI-1 in blood are associated with obesity, insulin resistance, diabetes and cardiovascular disease [1, 2, 3, 9, 10]. The concentrations of TG and FFA correlate positively with that of PAI-1 [1, 2, 5, 6, 9]. Agents that lower concentrations of TG lead to a decrease in the concentration of PAI-1 [5, 8, 17, 18, 19]. Our results demonstrated that both TG and FFA increase PAI-1 through diverse pathways.

Our previous results demonstrated that FFA augment expression of PAI-1 in HepG2 cells through induction of a transcription factor that binds to FFA response element located between –528 and –599 in the PAI-1 gene [16]. The present results extend the observations and demonstrate that protein kinase C is a critical component of the FFA signaling pathway. Furthermore, our results demonstrate that TG per se, rather than its constituent FFA alone, increases expression of PAI-1.

Our results with TG are consistent with those of Eriksson and colleagues demonstrating that VLDL augments expression of PAI-1 through a response element located between –672 and –657 in the PAI-1 gene [13]. Further, our results in combination with those of Eriksson et al suggest that TG is the moiety in VLDL predominately responsible for the effect of VLDL on the expression of PAI-1 [12, 14, 20] . In addition, the results are consistent with the observation that the TG content of LDL modulates expression of PAI-1 [20]. Nevertheless, the additive effect of FFA and TG demonstrated in the present studies suggest that the prevailing concentration of both TG and FFA contribute to the expression of PAI-1 in diabetic subjects.

Our results are consistent with those in our previous work in healthy human subjects in which infusion of Liposyn® and glucose increased expression of PAI-1 [11]. They explain, in part, the apparent discrepancy between results *in vivo* [21, 22] and *in vitro* [23, 24, 25] regarding the effect of insulin on expression of PAI-1. Thus, a reduction *in vivo* in the concentration of TG and FFA associated with infusion of insulin under euglycemic clamp techniques [11] would be expected to negatively affect PAI-1 expression. Accordingly, potentially positive effects of insulin would be offset by negative effects association with a reduction in the concentration of TG and FFA. Our results are consistent with the view that the combination of increased glucose, increased TG, increased FFA, and increased insulin and insulin precursors augment expression increase PAI-1 in patients with type 2 diabetes.

The insertion/deletion polymorphism of PAI-1 at –675 in the PAI-1 gene has been variably linked with altered expression of PAI-1 in healthy subjects and in patients with diabetes [15, 26, 27, 28]. As expected based on the locus of the FFA response element, similar effects were seen with either the 4G or 5G polymorphism in response to FFA. By contrast, the 4G/5G polymorphism is located in the TG response element. Consistent with this location we found that the 4G polymorphism conferred a modest increase in sensitivity to TG. These results are concordant with previous observations of TG effects on PAI-1 as well as the modest influence of the 4G/5G polymorphism [13, 15, 29].

Activation of protein kinase C mediates the effects of diverse agonists on the expression of PAI-1 [30, 31, 32, 33]. Protein kinase C contributes to the induction of PAI-1 by phorbol esters, TNF-alpha, and TGF-beta in diverse cell types [32, 34, 35, 36], including HepG2 cells [32, 37], mesangial cells [30, 38], and endothelial cells [31, 33, 34]. In the present studies we used both H-7, a broad based serine-threonine kinase inhibitor and Bisindolylmaleimide I, a highly selective cell-permeable protein kinase C inhibitor. The FFA-induced increased expression of PAI-1 was attenuated more than 90% by either agent. By contrast, the TG-induced increased expression of PAI-1 was inhibited only 30% by the protein kinase C specific inhibitor Bisindolylmaleimide I. Higher concentrations of the broad based kinase inhibitor, known to inhibit diverse serine-threonine kinases, inhibited the effect of TG. Thus, the results demonstrate that serine-threonine kinases mediate effects of both TG and FFA on expression of PAI-1. Hydrolysis of TG yields FFA. The limited inhibitory effect of the selective protein kinase C inhibitor on the increased expression of PAI-1 is consistent with FFA mediating a component of the TG-induced effect [18, 39, 40].

In summary, our results demonstrate that TG and FFA increase expression of PAI-1 through independent signaling pathways. The effects of FFA are mediated by protein kinase C and a FFA response region that is located between –528 and –599 in the PAI-1 gene. By contrast, the effects of TG are mediated by a distinct region (-672 to -657), influenced to a modest extent by the 4G/5G polymorphism, and induced by a serine-threonine kinase other than protein kinase C.

***Acknowledgement***: Supported by a grant from the American Diabetes Association (D.J. Schneider)

### REFERENCES

20. Allison, B.A., Nilsson, L., Karpe, F., Hamsten, A. and Eriksson, P. (1999) Effects of native, triglyceride-enriched, and oxidatively modified LDL on plasminogen activator inhibitor-1 expression in human endothelial cells. *Arterioscler. Thromb. Vasc. Biol.* **19**, 1354-1360.

8. Andersen, P., Smith, P., Seljeflot, I., Brataker, S. and Arnesen, H. (1990) Effects of gemfibrozil on lipids and haemostasis after myocardial infarction. *Thromb. Haemost.* **63**, 174-177.

17. Arts J., Kockx M., Princen H.M.G. and Kooistra T. (1997) Studies on the Mechanism of Fibrate-Inhibited Expression of Plasminogen Activator Inhibitor-1 in Cultured Hepatocytes From Cynomolgus Monkey. *Arterioscler Thromb Vasc Biol.* **17**, 26-32.

32. Arts, J., Grimbergen, J., Bosma, P.J., Rahmsdorf, H.J. and Kooistra, T. (1996) Role of c-Jun and proximal phorbol 12-myristate-13-acetate-(PMA)-responsive elements in the regulation of basal and PMA-stimulated plasminogen-activator inhibitor-1 gene expression in HepG2. *Eur. J. Biochem.* **241**, 393-402.

37. Banfi, C., Mussoni, L., Ris, P., Cattaneo, M.G., Vicentini, L., Battaini, F., Galli, C. and Tremoli, E. (1999) Very low density lipoprotein-mediated signal transduction and plasminogen activator inhibitor type 1 in cultured HepG2 cells. *Circ. Res.* **85**, 208-217.

19. Brown, S.L., Sobel, B.E. and Fujii, S. (1995) Attenuation of the synthesis of plasminogen activator inhibitor type 1 by niacin. A potential link between lipid lowering and fibrinolysis. *Circulation* **92**, 767-772.

11. Calles-Escandon, J., Mirza, S.A., Sobel, B.E. and Schneider, D.J. (1998) Induction of hyperinsulinemia combined with hyperglycemia and hypertriglyceridemia increases plasminogen activator inhibitor 1 in blood in normal human subjects. *Diabetes* **47**, 290-293.

16. Chen, Y., Billadello, J.J. and Schneider, D.J. (2000) Identification and localization of a fatty acid response region in the human plasminogen activator inhibitor-1 gene. *Arterioscler. Thromb. Vasc. Biol.* **20**, 2696-2701.

27. Dawson, S.J., Wiman, B., Hamsten, A., Green, F., Humphries, S. and Henney, A.M. (1993) The two allele sequences of a common polymorphism in the promoter of the plasminogen activator inhibitor-1 (PAI-1) gene respond differently to interleukin-1 in HepG2 cells. *J. Biol. Chem.* **268**, 10739-10745.

14. Dichtl, W., Ares, M.P., Stollenwerk, M., Giachelli, C.M., Scatena, M., Hamsten, A., Eriksson, P. and Nilsson, J. (2000) In vivo stimulation of vascular plasminogen activator inhibitor-1 production by very low-density lipoprotein involves transcription factor binding to a VLDL-responsive element. *Thromb. Haemost.* **84**, 706-711.

15. Eriksson, P., Kallin, B., van 't, H., Bavenholm, P. and Hamsten, A. (1995) Allele-specific increase in basal transcription of the plasminogen-activator inhibitor 1 gene is associated with myocardial infarction. *Proc. Natl. Acad. Sci. U.S.A.* **92**, 1851-1855.

13. Eriksson, P., Nilsson, L., Karpe, F. and Hamsten, A. (1998) Very-low-density lipoprotein response element in the promoter region of the human plasminogen activator inhibitor-1 gene implicated in the impaired fibrinolysis of hypertriglyceridemia. *Arterioscler. Thromb. Vasc. Biol.* **18**, 20-26.

34. Fukao, H., Matsumoto, H., Ueshima, S., Okada, K. and Matsuo, O. (1995) Effects of fibrin on the secretion of plasminogen activator inhibitor-1 from endothelial cells and on protein kinase C. *Life Sci.* **57**, 1267-1276.

22. Grant, P.J., Kruithof, E.K., Felley, C.P., Felber, J.P. and Bachmann, F. (1990) Short-term infusions of insulin, triacylglycerol and glucose do not cause acute increases in plasminogen activator inhibitor-1 concentrations in man. *Clin. Sci.(Colch.)* **79**, 513-516.

26. Gray, R.P., Yudkin, J.S. and Patterson, D.L. (1993) Enzymatic evidence of impaired reperfusion in diabetic patients after thrombolytic therapy for acute myocardial infarction: a role for plasminogen activator inhibitor? *Br.Heart J.* **70**, 530-536.

36. Halstead, J., Kemp, K. and Ignotz, R.A. (1995) Evidence for involvement of phosphatidylcholine-phospholipase C and protein kinase C in transforming growth factor-beta signaling. *J. Biol. Chem.* **270**, 13600-13603.

1. Juhan-Vague I., Alessi M.C. and Vague P. (1991) Increased plasma plasminogen activator inhibitor 1 levels. A possible link between insulin resistance and atherosthrombosis. *Diabetologia* **34**, 457-462.

2. Juhan-Vague I., Vague P., Alessi M.C., Badier C., Valadier J., Aillaud M.F. and Atlan C. (1987) Relationships Between Plasma Insulin Triglyceride, Body Mass Index and Plasminogen Activator Inhibitor 1. *Diabetes & Metabolism* **13**, 331-336.

7. Kaneko, T., Wada, H., Wakita, Y., Minamikawa, K., Nakase, T., Mori, Y., Deguchi, K. and Shirakawa, S. (1994) Enhanced tissue factor activity and plasminogen activator inhibitor-1 antigen in human umbilical vein endothelial cells incubated with lipoproteins. *Blood Coagul. Fibrinolysis* **5**, 385-392.

9. Keber I and Keber D (1992) Increased Plasminogen Activator Inhibitor Activity in Survivors of Myoardial Infarction Is Associated with Metabolic Risk Factors of Atherosclerosis. *Haemostasis* **22**, 187-194.

33. Kooistra, T., Bosma, P.J., Toet, K., Cohen, L.H., Griffioen, M., van den Berg, E., le Clercq, L. and van, H., V (1991) Role of protein kinase C and cyclic adenosine monophosphate in the regulation of tissue-type plasminogen activator, plasminogen activator inhibitor-1, and platelet-derived growth factor mRNA levels in human endothelial cells. Possible involvement of proto-oncogenes c-jun and c-fos. *Arterioscler. Thromb.* **11**, 1042-1052.

25. Le Magueresse-Battistoni, B., Pernod, G., Kolodie, L., Morera, A.M. and Benahmed, M. (1997) Tumor necrosis factor-alpha regulates plasminogen activator inhibitor-1 in rat testicular peritubular cells. *Endocrinology* **138**, 1097-1105.

29. Li, X.N., Grenett, H.E., Benza, R.L., Demissie, S., Brown, S.L., Tabengwa, E.M., Gianturco, S.H., Bradley, W.A., Fless, G.M. and Booyse, F.M. (1997) Genotype-specific transcriptional regulation of PAI-1 expression by hypertriglyceridemic VLDL and Lp(a) in cultured human endothelial cells. *Arterioscler. Thromb. Vasc. Biol.* **17**, 3215-3223.

3. McGill, J.B., Schneider, D.J., Arfken, C.L., Lucore, C.L. and Sobel, B.E. (1994) Factors responsible for impaired fibrinolysis in obese subjects and NIDDM patients. *Diabetes* **43**, 104-109.

10. Mehta, J., Mehta, P., Lawson, D. and Saldeen, T. (1987) Plasma tissue plasminogen activator inhibitor levels in coronary artery disease: correlation with age and serum triglyceride concentrations. *J. Am. Coll. Cardiol.* **9**, 263-268.

38. Motojima, M., Ando, T. and Yoshioka, T. (2000) Sp1-like activity mediates angiotensin-II-induced plasminogen-activator inhibitor type-1 (PAI-1) gene expression in mesangial cells. *Biochem. J.* **349**, 435-441.

30. Motojima, M., Kakuchi, J. and Yoshioka, T. (1999) Association of TGF-beta signaling in angiotensin II-induced PAI-1 mRNA upregulation in mesangial cells: role of PKC. *Biochim. Biophys. Acta* **1449**, 217-226.

5. Mussoni l., Mannucci L., Sirtori M., Camera M., Maderna P., Sironi L. and Tremoli E. (1992) Hypertriglyceridemia and regulation of fibrinolytic activity. *Arterioscler Thromb.* **12**, 19-27.

40. Nilsson, L., Banfi, C., Diczfalusy, U., Tremoli, E., Hamsten, A. and Eriksson, P. (1998) Unsaturated fatty acids increase plasminogen activator inhibitor-1 expression in endothelial cells. **Arterioscler.** Thromb.Vasc. Biol. **18**, 1679-1685.

18. Nilsson, L., Gafvels, M., Musakka, L., Ensler, K., Strickland, D.K., Angelin, B., Hamsten, A. and Eriksson, P. (1999) VLDL activation of plasminogen activator inhibitor-1 (PAI-1) expression: involvement of the VLDL receptor. *J. Lipid Res.* **40**, 913-919.

25. Nordt, T.K., Sawa, H., Fujii, S. and Sobel, B.E. (1995) Induction of plasminogen activator inhibitor type-1 (PAI-1) by proinsulin and insulin in vivo. *Circulation* **91**, 764-770.

23. Schneider, D.J., Absher, P.M. and Ricci, M.A. (1997) Dependence of augmentation of arterial endothelial cell expression of plasminogen activator inhibitor type 1 by insulin on soluble factors released from vascular smooth muscle cells. *Circulation* **96**, 2868-2876.

24. Schneider, D.J. and Sobel, B.E. (1991) Augmentation of synthesis of plasminogen activator inhibitor type 1 by insulin and insulin-like growth factor type I: implications for vascular disease in hyperinsulinemic states [published erratum appears in Proc Natl Acad Sci U S A 1992 Feb 1;89(3):1148]. *Proc. Natl. Acad. Sci. U.S.A.* **88**, 9959-9963.

12. Schneider, D.J. and Sobel, B.E. (1996) Synergistic augmentation of expression of plasminogen activator inhibitor type-1 induced by insulin, very-low-density lipoproteins, and fatty acids. *Coron. Artery Dis.* **7**, 813-817.

6. Sironi L., Mussoni l., Prati L., Baldassarre D., Camera M., Banfi C. and Tremoli E. (1996) Plasminogen activator inhibitor type-1 synthesis and mRNA expression in HepG2 cells are regulated by VLDL. *Arterioscler Thromb Vasc Biol.* **16**, 89-96.

4. Sobel, B.E. (1999) Increased plasminogen activator inhibitor-1 and vasculopathy. A reconcilable paradox [editorial]. *Circulation* **99**, 2496-2498.

39. Stiko-Rahm, A., Wiman, B., Hamsten, A. and Nilsson, J. (1990) Secretion of plasminogen activator inhibitor-1 from cultured human umbilical vein endothelial cells is induced by very low density lipoprotein. *Arteriosclerosis* **10**, 1067-1073.

21. Vuorinen-Markkola, H., Puhakainen, I. and Yki-Jarvinen, H. (1992) No evidence for short-term regulation of plasminogen activator inhibitor activity by insulin in man. *Thromb. Haemost.* **67**, 117-120.

28. Ye, S., Green, F.R., Scarabin, P.Y., Nicaud, V., Bara, L., Dawson, S.J., Humphries, S.E., Evans, A., Luc, G. and Cambou, J.P. (1995) The 4G/5G genetic polymorphism in the promoter of the plasminogen activator inhibitor-1 (PAI-1) gene is associated with differences in plasma PAI-1 activity but not with risk of myocardial infarction in the ECTIM study. Etude CasTemoins de I'nfarctus du Mycocarde. *Thromb. Haemost.* **74**, 837-841.

31. Zidovetzki, R., Wang, J.L., Kim, J.A., Chen, P., Fisher, M. and Hofman, F.M. (1999) Endothelin-1 enhances plasminogen activator inhibitor-1 production by human brain endothelial cells via protein kinase C-dependent pathway. *Arterioscler. Thromb. Vasc. Biol.* **19**, 1768-1775.

